# Intelligent laparoscopic grasper with hybrid neural networks for real-time vascular detection in minimally invasive surgery

**DOI:** 10.1117/1.JBO.30.9.097001

**Published:** 2025-09-16

**Authors:** Pingping Wang, Yuting Huang, Chang Liu, Ziying Huang, Yuxin Huang, Yuan Wu, Zhengying Wang, Kaichao Chen, Zhengyong Liu, Dongxian Peng

**Affiliations:** a Zhujiang Hospital, Southern Medical University, Obstetrics and Gynecology Center, Department of Gynecology, Guangzhou, China; b Sun Yat-Sen University, School of Electronics and Information Technology, Guangdong Provincial Key Laboratory of Optoelectronic Information Processing Chips and Systems, Guangzhou, China; c Southern Medical University, School of Biomedical Engineering, Guangzhou, Guangdong, China

**Keywords:** fiber Bragg grating, convolutional neural network combined with long short-term memory algorithm, blood vessel identification, minimally invasive surgery

## Abstract

**Significance:**

We address the challenge of inadequate force feedback in laparoscopic surgery, which increases the risk of vessel injury. By integrating fiber Bragg grating (FBG) sensors with a laparoscopic grasper and employing a convolutional neural network combined with long short-term memory (CNN-LSTM) algorithm, this approach enables real-time, accurate vessel identification, potentially reducing surgical complications.

**Aim:**

Laparoscopic surgery is often hindered by inadequate force feedback, especially in complex scenarios involving tumor invasion and pelvic-abdominal adhesion, leading to challenges in locating blood vessels and an increased risk of vessel injury. Thus, it is desirable to develop a laparoscopic system capable of distinguishing the location and type of the vessels during surgery, which requires a compact and highly sensitive sensor integrated with a laparoscopic grasper.

**Approach:**

We present an innovative laparoscopic grasper integrated with FBG force sensors for real-time force feedback, employing silicone and porcine vessel models to simulate varying depths and tissue coverage. The device successfully captured specific vessel signals, which were processed through a CNN-LSTM algorithm, enabling real-time vessel identification in minimally invasive surgery (MIS).

**Results:**

The intelligent laparoscopic grasper successfully obtained distinct vessel signals under varying conditions. As a result, the mean vessel gripping force for porcine vessel model III was 0.059 N under fatty tissue and 0.032 N under muscle tissue (p<0.001). The CNN-LSTM algorithm achieved a precision of 97.06% in vessel identification across different tissue coverages.

**Conclusions:**

The FBG sensor-integrated laparoscopic grasper, assisted by the processing of the CNN-LSTM algorithm, demonstrated the ability to identify vessels *ex vivo* across different models. This technology holds potential for real-time and accurate vessel identification during MIS, which could significantly reduce the occurrence of unnecessary vessel injuries.

## 1 Introduction

Minimally invasive surgery (MIS) has gained favor among both doctors and patients due to its advantages of smaller incisions, faster postoperative recovery, and shorter hospital stays.^1^^,^^2^ However, compared with traditional open abdominal surgery, MIS suffers from a lack of force feedback, and in certain cases, this feedback may be entirely absent.^3^^,^^4^ As the utilization of MIS expands, surgeons increasingly encounter challenges such as tissue adhesion, tumor invasion, and obesity, which significantly elevate the difficulty of identifying blood vessels during procedures and increase the risk of unintended vascular damage.^5^ In a review of reported literature, the incidence of complications in laparoscopic surgery ranges between 1.49% and 31.58%,^6^^,^^7^ with vascular injuries representing the most severe complications, accounting for 30% to 64% of all complications.^8^^,^^9^ These figures indicate a considerable risk although the actual probability of vascular injuries during surgery may be even higher. Intraoperative vascular injuries can obstruct the surgical field of vision, increasing the likelihood of conversion to open surgery, and severe vascular injuries can lead to tissue ischemic necrosis, hemorrhagic shock, and even death in patients.^10^ The occurrence of such serious complications poses a substantial challenge to the implementation and widespread adoption of MIS, highlighting the importance of blood vessel identification during MIS.

To address the challenge of precise vessel techniques in MIS, various technological innovations have been proposed in recent years. The most extensive research has been conducted on vessel identification technologies based on imaging technology. Fluorescence imaging, for instance, identifies blood vessels by injecting a fluorescent agent, but it may cause allergic reactions and usually needs complex equipment for imaging, which is not easy to achieve during the operation of MIS.^11^^,^^12^ Ultrasound imaging is noninvasive and cost-effective, yet its effectiveness heavily depends on the operator’s skill.^13^ CT and MRI offer high resolution but are not suitable for real-time surgical navigation.^14^ Near-infrared imaging has strong penetration capabilities for deep tissues but is limited by its resolution.^15^ Each of these imaging technologies has its own merits for vessel identification but also presents limitations in complex MIS scenarios, such as low resolution, slow identification speed, and integration challenges with minimally invasive surgical instruments. These deficiencies significantly hinder the practical application of imaging technology for vascular identification during MIS.

Recently, the use of sensors to monitor and identify vascular parameters based on the pulsation and rhythmicity of blood vessels has attracted significant attention. Prior investigations have been conducted on vascular identification in extracorporeal vascular models utilizing the cyclical contraction and relaxation of blood vessels, which cause changes in resistance, voltage, and current parameters. For instance, Beasle et al.^16^ employed a capacitive sensor technology to differentiate various courses of blood vessels on silicone models. Jung et al.^17^ utilized piezoresistive sensors to identify blood vessels of different depths and diameters on silicone models. However, these studies remain at the experimental stage with extracorporeal vascular models, and the low biosafety associated with electrically related sensors significantly limits their application in MIS.

Optical fiber sensors, due to their small size, high flexibility, biocompatibility, and resistance to electromagnetic interference, hold great promise for medical applications and have been widely adopted across various medical fields.^18^^,^^19^ In research related to vascular identification, Ahmadi et al.^20^ utilized a microstructure optical fiber-based sensing technology to identify simulated arteries and tumors, although the study was confined to extracorporeal model exploration. Ho et al.^21^ developed a puncture needle embedded with FBG sensors and tested it on vascular models, yielding significant findings for artery identification. Nevertheless, this technology is constrained by its invasiveness and reduced effectiveness in identifying veins. Li et al.^22^ prepared FBG sensors using multiple single-core fiber gratings to identify tissues with varying hardness, establishing static vascular models for their experiments, which however was restricted to vascular models and no in-depth research has been conducted. In this paper, we propose and demonstrate a laparoscopic grasper integrated with FBG tactile force sensors to achieve the identification of tissue vessels, assisted by a data-processing method of deep learning. The force sensors are encapsulated within the laparoscopic grasper, allowing for the detection of blood vessels without interfering with the surgical clamping operation, unlike other sensors.^23^^,^^24^ To validate the performance of the system, various phantom and *ex vivo* animal experiments were conducted, and the experimental results confirmed the high efficacy of the proposed instrument in detecting blood vessels at certain locations.

## 2 Materials and Methods

### 2.1 Preparation of Sensing System and Blood Vessels

#### 2.1.1 Real-time force feedback system

**Fig. 1:**
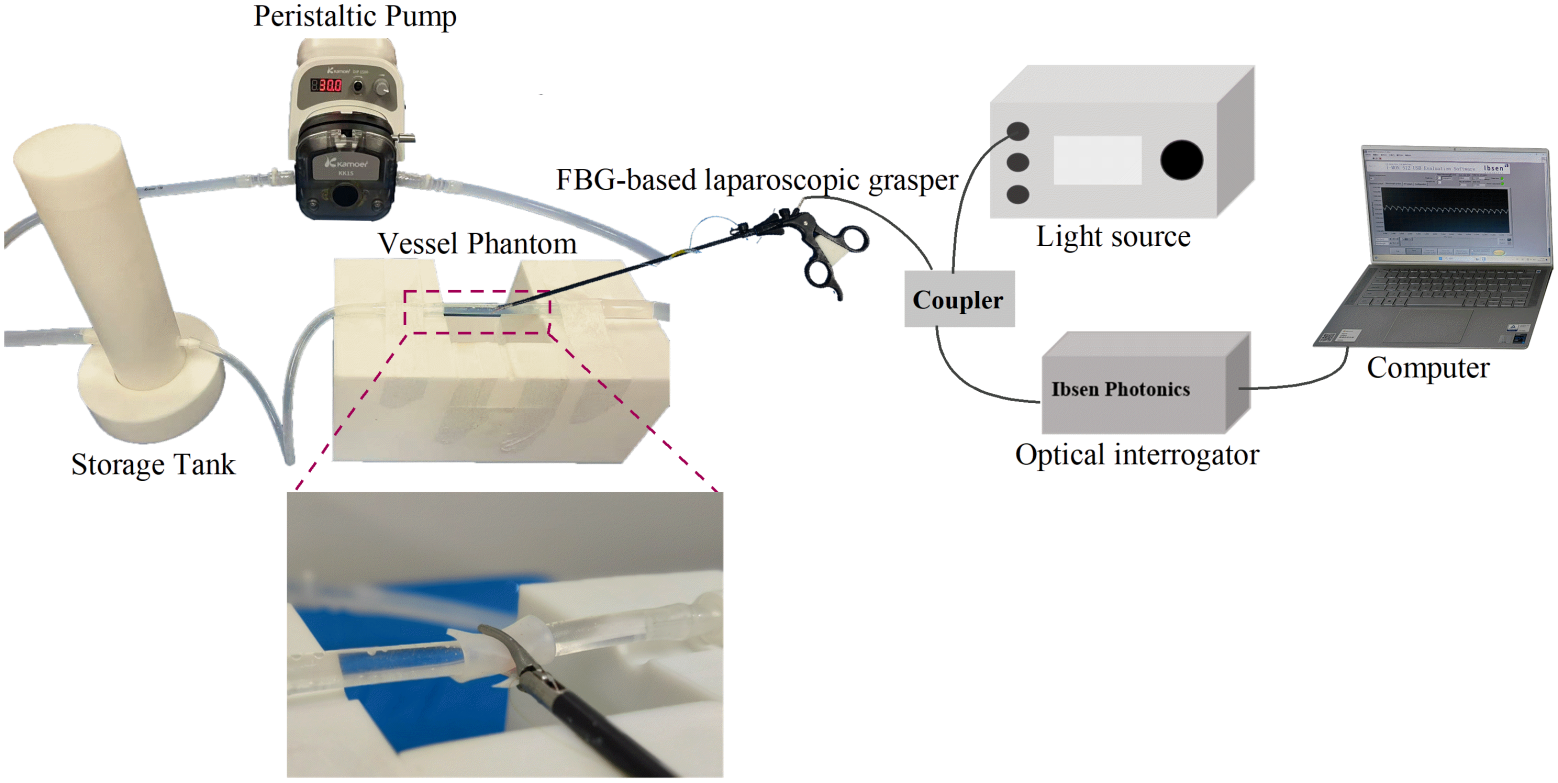
Proposed real-time force feedback system and the blood vessel system.

As illustrated in Fig. 1, a real-time force feedback system was developed for the experimental investigation. The system consists of an intelligent laparoscopic grasper integrated with an FBG-based tactile sensor, an optical interrogator, a light source, and a personal computer for displaying and recording the real-time gripping force data. The FBG-based tactile sensor, which is fixed at the tip of the grasper, exhibits a strain sensitivity of 0.082  nm/N. In principle, FBG is such a device that reflects a specific wavelength of broadband light known as the Bragg wavelength $\lambda_{B}$, which is determined by the effective index of refraction ($n_{\mathrm{eff}}$) of the fundamental mode propagating in the fiber core and the grating period (Λ), as expressed by Eq. (1). The application of force to the FBG results in grating deformation and a change in the refractive index, leading to a corresponding shift in the Bragg wavelength $\lambda_{B}$, as defined by Eq. (2), where $P_{e}$ is photo-elastic coefficient, η is a factor of force transferred to strain, and F is the applied force by the tissue vessels

$$\lambda_{B}=2n_{\mathrm{eff}}\Lambda ,$$ (1)

$$\Delta \lambda_{B}=2n_{\mathrm{eff}}\Lambda (1+P_{e})\cdot (\eta F).$$ (2)

#### 2.1.2 Blood vessel system

##### Test phantom creation

As illustrated in Fig. 1, the blood flow simulation system employed in this study comprises a peristaltic pump (DIP 1500 V2, Kamoer, Shanghai, China), a vessel phantom, and a fluid storage tank. The peristaltic pump maintains a constant flow rate by pumping fluid through a tube, mimicking the action of the human heart. This is achieved by alternately compressing and releasing the silicone blood vessel using three rollers, which produces a pulsatile fluid flow. The vessel phantom is composed of silicone tubes and silicone sheets. The silicone tubes vary in diameter, flexibility, and wall thickness to simulate different types of blood vessels. Silicone sheets with different stiffness and thickness levels simulate surrounding tissues, such as adipose tissue, scar tissue, and tumor tissue, which are set as arteries or veins under varying physiological conditions. The identification and ligation of the uterine artery are critical steps in hysterectomy procedures, with the average diameter of the uterine artery typically around 5.0 mm. To design a vessel model that closely mimics this artery, the blood vessel phantoms (China Ningbo Chuangdao 3D Medical Technology Co., Ltd.) were fabricated from biomedical-grade polyurethane silicone, with hardness levels of 20A, 30A, and 40A (diameter = 5.0 mm, wall thickness = 1.0 mm). In addition, three types of silicone gels (Smooth-On, Inc. Ecoflex 00-10/00-20/00-30) with hardness levels of 10A, 20A, and 30A were used to simulate the surrounding tissues. The fluid storage tank ensures continuous circulation of the fluid medium. To simulate various vessel environments, three conditions were established using silicone-based tissues of varying hardness and thickness surrounding the vessels. In vessel phantom I, silicone tubes with stiffness levels of 20A, 30A, and 40A were pulsated without surrounding silicone gel. In vessel phantom II, a 30A silicone tube was surrounded by phantom tissue with a hardness of 10A, with varying thicknesses of 0.5, 1.0, 1.5, 2.0, 2.5, and 3.0 mm. In vessel phantom III, a 30A silicone tube was surrounded by 1.0 mm thick phantom tissue with hardness levels of 10A, 20A, and 30A.^25^^,^^26^

##### *Ex vivo* porcine vessels

**Fig. 2:**
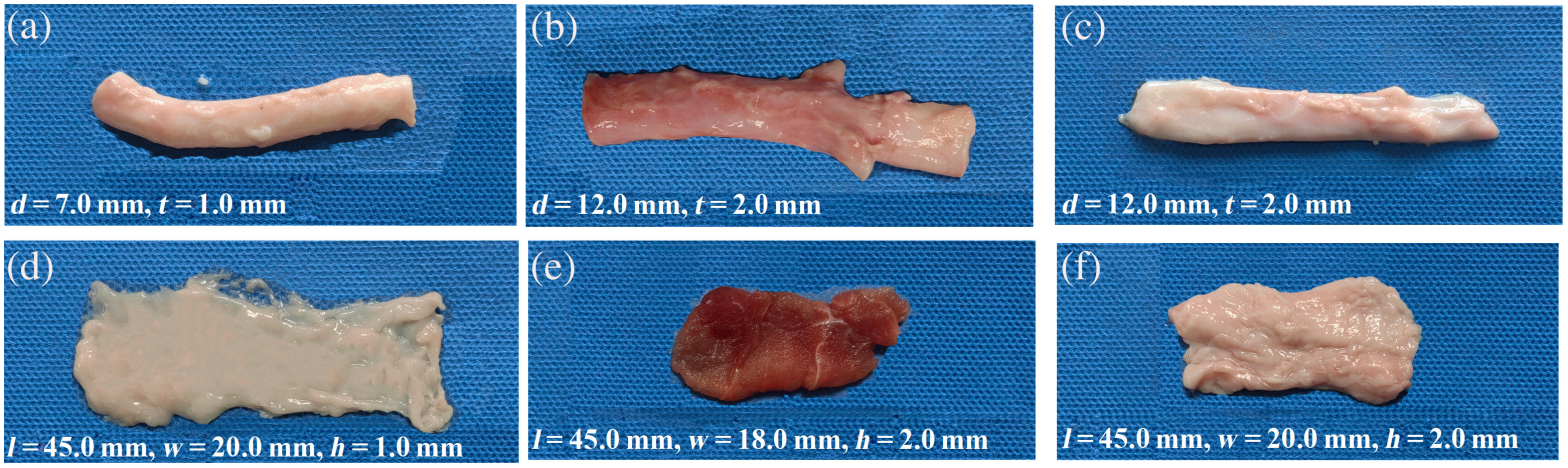
Upper section of the figure presents illustrative images depicting the diameter (d) and wall thickness (t) of porcine blood vessels. (a) Porcine carotid artery, d=7.0  mm, t=1.0  mm. (b) Abdominal artery, d=12.0  mm, t=2.0  mm. (c) Abdominal veins, d=12.0  mm, t=2.0  mm. The lower section of the figure displays the dimensions, including length (l), width (w), and height (h) of the surrounding tissues. (d) Porcine adipose tissues, l=45.0  mm, w=20.0  mm, and h=1.0  mm. (e) Porcine musculature, l=45.0  mm, w=18.0  mm, and h=2.0  mm. (f) Porcine adipose tissues, l=45.0  mm, w=20.0  mm, and h=2.0  mm.

To conduct further investigation, an *ex vivo* experiment was performed utilizing porcine vessels and tissues. Prior studies have demonstrated that porcine tissues closely resemble human tissues in physiological characteristics. The blood vessels and tissues were obtained from six Landrace porcine animals (n=6), each with an approximate weight of 80 kg, sourced from Guangzhou Yushan Meat Food Co, Ltd. (Dashi 4A Central Slaughterhouse). These tissues included porcine carotid arteries, abdominal arteries, veins, musculature, and adipose tissues, which are preserved in saline solution immediately after excision to maintain freshness. The dimensions of these vessels and tissues are shown in Fig. 2. In porcine vessel model I, the abdominal artery and vein were set up to pulsate without any surrounding tissue to simulate pulsations of blood vessels with varying degrees of hardness. In porcine vessel model II, the porcine carotid artery was surrounded by adipose tissue with thicknesses of 1.0, 2.0, and 3.0 mm, individually. In porcine vessel model III, the porcine carotid artery was encircled by both porcine musculature and adipose tissues to mimic the complexity of *in vivo* conditions.

### 2.2 Experimental Protocol

**Fig. 3:**
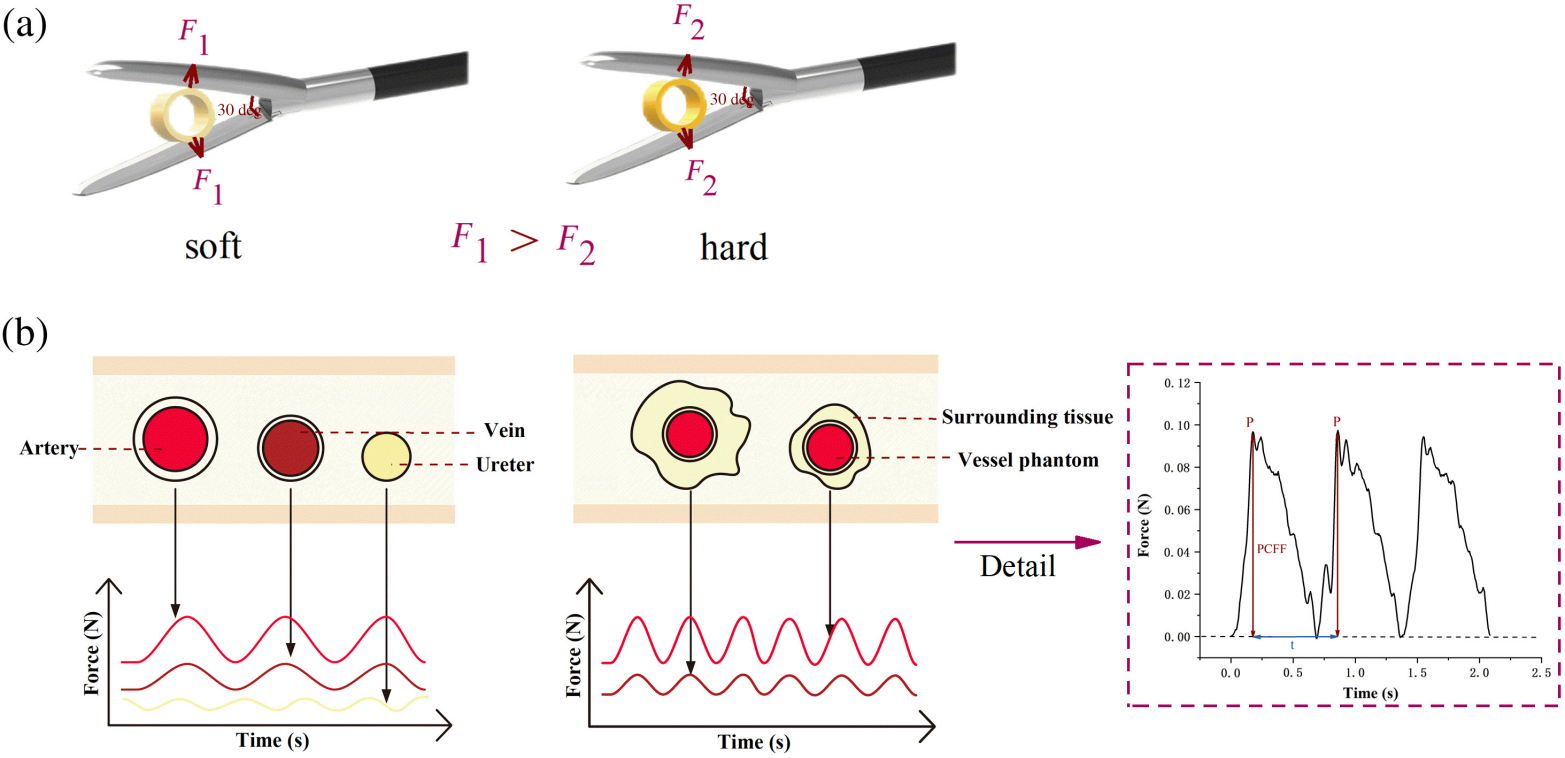
Experimental principle. (a) A consistent clamping angle causes different perceptions of pulsation force depending on the hardness of the pipe wall. (b) Diagram illustrating the variation in force waveforms when gripping blood vessels with different tissue types.

To mimic the vascular pulsation under normal physiological conditions, the working frequency of the peristaltic pump was set to 30 revolutions/min, corresponding to a simulated heart rate of ∼90  beats/min in our experiment. The laparoscopic grasper was initiated in a fully open position and gradually closed until it reached a grasping angle of 30 deg and then maintained. Once the signal got stabilized, this position was held steady for 15 s. The entire experiment was repeated 80 times, recording data on vessel pulsations under different conditions. Figure 3 depicts the experimental principle, highlighting that consistent clamping angle results in decreased pulsation force perception within the pipe as the pipe wall hardness increases. Specifically, we maintained the clamping angle of 30 deg and observed that as the blood vessel wall became harder, the pulsation force detected by the sensor decreased. In addition, blood vessels at the same depth but surrounded by different tissues exhibited unique waveforms.

The gripping waveform in this study comprises an ascending and descending phase. Rapid fluid expulsion increases the silicone blood vessel wall’s internal pressure, causing the ascending phase to steepen and peak at maximum fluid pressure. In this study, the peak force measured by the FBG-based sensor is referred to as the peak clamping force fluctuation (PCFF). The PCFF provides insights into the dynamic interaction between the grasper and the blood vessel. The frequency of pulsations is calculated based on the distance between the PCFF, reflecting the vascular response under the simulated physiological conditions.

The morphological characteristics of the gripping force waveform produced by the sensor are closely related to the hemodynamic behavior of the circulatory system. When clamping a target blood vessel at a fixed angle with stable hemodynamic parameters, the detected waveform of the clamping force is primarily shaped by the inherent properties of the blood vessel and the surrounding tissue. Thus, the PCFF assesses the blood vessel or surrounding tissue’s basic condition, whereas the distance between the peaks of clamping force can be used to calculate the pulse frequency.

### 2.3 Statistical Analysis

The independent sample t-test is employed for comparing means between two groups when data adhere to a normal distribution with equal variance. If the normality is violated, the nonparametric Mann–Whitney U test is utilized to compare medians. For analysis involving three or more independent groups where parametric assumptions are not met, the nonparametric Kruskal–Wallis test is used to assess differences in experimental group medians. Typically, a P-value of less than 0.05 is considered statistically significant for inter-group differences.^27^ All statistical analyses were conducted using SPSS 26.0 (IBM, New York, United States).

### 2.4 Neural Network Model and Vessel Recognition

In the experiment, the FBG sensor measured a substantial amount of sampling data for each group, which requires comprehensive analysis and processing to enable real-time vessel identification. To achieve precise and timely blood vessel identification, a convolutional neural network combined with a long-short term memory (CNN-LSTM) algorithm is proposed and constructed to process the signals recorded by an FBG tactile force sensor on the laparoscopic grasper.

#### 2.4.1 Preprocessing of the measured force signal

The preprocessing of data includes denoising, normalization, and smoothing operations. Specifically, we implemented wavelet thresholding for signal denoising and subsequently used the Z-score method for signal normalization. Wavelet threshold denoising is preferred for its ability to preserve edges in nonstationary force signals. Its multiscale decomposition capability effectively handles the simultaneous presence of transient pulses and low-frequency deformations commonly encountered during surgery. The wavelet threshold denoising process involves segmenting the signal into various wavelet sub-bands through wavelet transformation. A soft threshold function is utilized to compare the wavelet coefficients of each sub-band against a threshold value, below which the coefficients are deemed as interfering noise and consequently discarded. The signal is then reconstructed using a wavelet reconstruction function, effectively mitigating noise levels. Next, we standardized the input sequence by subtracting the mean and dividing by the standard deviation, unifying data of disparate magnitudes to the same scale and ensuring comparability across the dataset. Furthermore, a moving average filter with a window width of 3 was incorporated to smooth the output, enhancing data consistency and minimizing variability. Ultimately, the dataset was partitioned into training, validation, and test datasets in a ratio of 6:2:2. The algorithm processes one-dimensional force signals as input, analyzing sampled data points within a short timeframe and outputting the recognition results.

#### 2.4.2 Configuration of CNN-LSTM

**Fig. 4:**
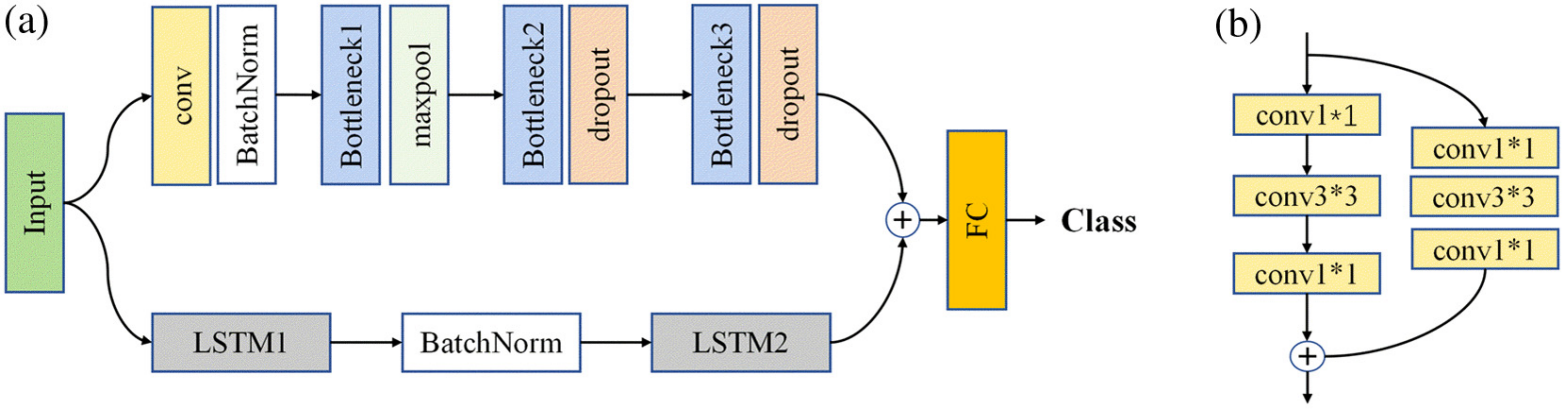
(a) Schematic structure of the proposed CNN-LSTM network, (b) structure of the bottleneck block used in the network.

The neural network model introduced in this work employs a CNN-LSTM architecture, as illustrated in Fig. 4(a). The CNN-LSTM model consists of four CNN layers and three LSTM layers, arranged in two distinct paths. The outputs from these two paths are merged into a fully connected (FC) layer, with a final output that provides the classification result with a number of output classes adjusted to the specific classification task. Within the CNN pathway, the first layer consists of a convolutional layer, followed by batch normalization. The second layer comprises a bottleneck block and max pooling, whereas the subsequent two layers are composed of bottleneck blocks accompanied by dropout. As illustrated in Fig. 4(b), the bottleneck block consists of a main trunk and a side branch, both of which include a sequence of 1×1, 3×3, and 1×1 convolutional layers. The first 1×1 convolutional layer reduces the channel dimensions. The subsequent 3×3 convolutional layer is crucial for the bottleneck as it captures fine-grained features within the signal. The third 1×1 convolutional layer expands the channel dimensions back to their original size. The final output is obtained by summing the convolutional outputs from the trunk and the branch, thereby completing the residual structure. All units are activated by a rectified linear unit (ReLU) function.

In the LSTM pathway, two LSTM layers and a batch normalization layer are combined to extract temporal features. Initially, the data are fed into an LSTM layer to generate a 64-dimensional temporal feature vector, which is then normalized through a batch normalization layer and processed through another LSTM layer for dimensionality reduction, yielding an output of 20 values. Each LSTM layer includes a random dropout layer to enhance the model’s generalization capability. The local features extracted by the CNN and the temporal features from the LSTM are concatenated. These combined features are then input into a fully connected layer to produce the final classification result. The network is trained using stochastic gradient descent with a Nesterov momentum of 0.9 and a learning rate of 0.002. A cosine annealing schedule with restarts is employed for learning rate adjustment, with alpha set to 0 and 50 decay steps, and the learning rate is multiplied by 2 at each restart. The loss function used is binary cross-entropy, which is optimized for sequence prediction tasks. The model was implemented using the PyTorch framework on a workstation equipped with six RTX 2080 Ti GPUs.

A comprehensive spatiotemporal feature analysis of the CNN-LSTM dual-branch design reveals that the CNN branch extracts local morphological features from vascular contacts, whereas the LSTM branch models long-range dependencies, such as pulsation rhythms. The integration of these two pathways enhances the system’s ability to accurately distinguish among different states of blood vessels. Hyperparameters were optimized through grid search, choosing a learning rate of 0.002 and Nesterov momentum of 0.9 to speed up convergence and reduce oscillations, on the basis of the Adam method.^28^ The initial values of parameters were chosen for searching over a dense grid and faster convergence, which were then updated during training. Nesterov momentum helps stabilize noisy surgical force data by calculating gradients before updates. Cosine annealing with restarts (alpha = 0, 50 decay steps) was used to avoid local minima and speed up convergence. According to the raw data characteristics, a batch size of 128 was selected to optimize GPU use (6×RTX 2080 Ti), with larger batches reducing generalization and smaller ones causing instability. Binary cross-entropy was chosen as the loss function for its effectiveness with imbalanced classes in surgical data.^29^^,^^30^

## 3 Results

### 3.1 Performance in Detecting Silicone Tube in Phantoms

As presented in Tables 1–3, the mean PCFF values for vessel phantom I, which comprised vessels with hardness levels of 20A, 30A, and 40A, were 0.162 N, 0.130 N, and 0.010 N, respectively. In vessel phantom II, where gripping silicone blood vessels were encircled by tissues with thicknesses ranging from 0.5 to 3.0 mm in a step of 0.5 mm, the mean PCFF values were 0.223 N, 0.172 N, 0.141 N, 0.081 N, 0.050 N, and 0.026 N. For vessel phantom III, the mean PCFF values for silicone blood vessels encircled by silicone tissues with hardness levels of 10A, 20A, and 30A were 0.141 N, 0.100 N, and 0.058 N, respectively. The mean PCFF of blood vessels covered by tissues of varying hardness differs significantly (p<0.001), indicating that the FBG-based sensor can identify blood vessels covered by surrounding tissues of different hardness. Similar results were observed in vessel phantom I and vessel phantom II.

**Table 1:** Comparison of PCFF values of vessel phantom I.

Hardness of silicone vessel	The mean of PCFF (N)	VS 30A (H value)	VS 40A (H value)
20A	0.162	89.466^***^	162.966^***^
30A	0.130	—	74.52^***^
40A	0.010	—	—

*p<0.05; **p<0.01; ***p<0.00.

**Table 2:** Comparison of PCFF values of vessel phantom II.

Thickness of surrounding tissues on vessel (mm)	Mean of PCFF (N)	VS1.0 mm (H value)	VS 1.5 mm (H value)	VS 2.0 mm (H value)	VS 2.5 mm (H value)	VS 3.0 mm (H value)
0.5	0.223	91.400^**^	175.226^***^	269.267^***^	354.129^***^	441.663^***^
1.0	0.172	—	83.826^**^	177.867^***^	262.729^***^	350.263^***^
1.5	0.141	—	—	94.042^**^	178.904^***^	266.437^***^
2.0	0.081	—	—	—	84.862^**^	172.396^***^
2.5	0.050	—	—	—	—	87.534^**^
3.0	0.026	—	—	—	—	—

*p<0.05; **p<0.01; ***p<0.001.

**Table 3:** Comparison of PCFF values of vessel phantom III.

Hardness of surrounding tissues on vessel	Mean of PCFF (N)	VS 20A (H value)	VS 30A (H value)
10A	0.141	77.16^***^	153.83^***^
20A	0.100	—	76.67^***^
30A	0.058	—	—

*p<0.05; **p<0.01; ***p<0.001.

**Fig. 5:**
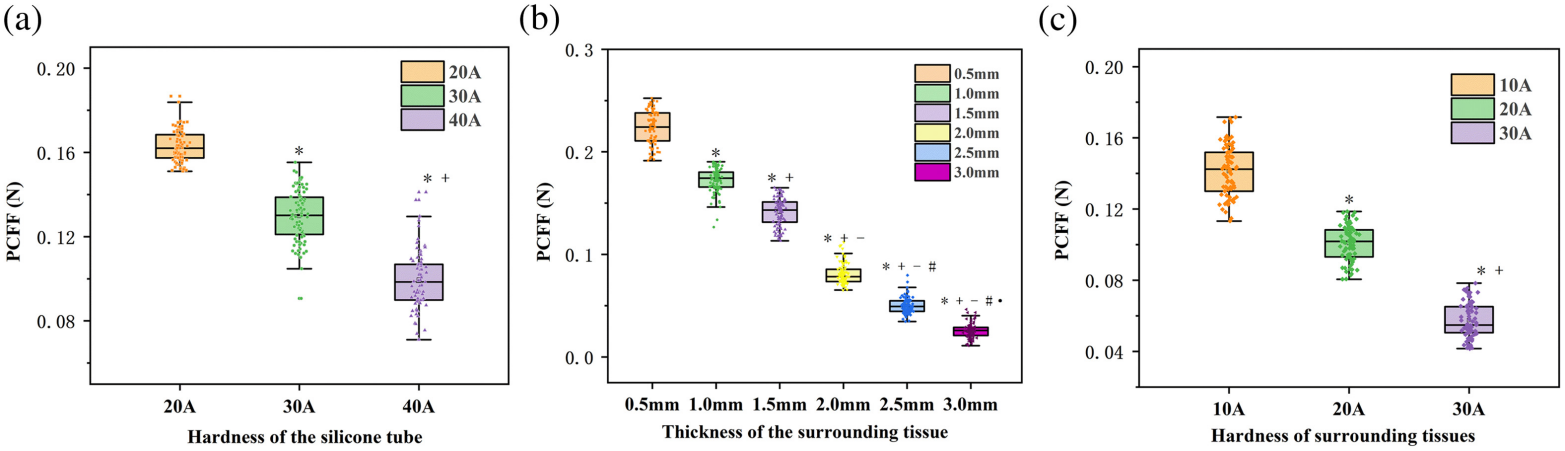
Box plots for vessel phantoms I to III. (a) Silicone blood vessels with different vessel hardness. (b) Surrounded by tissues of varying thicknesses. (c) Surrounded by tissues of varying hardness.

**Fig. 6:**
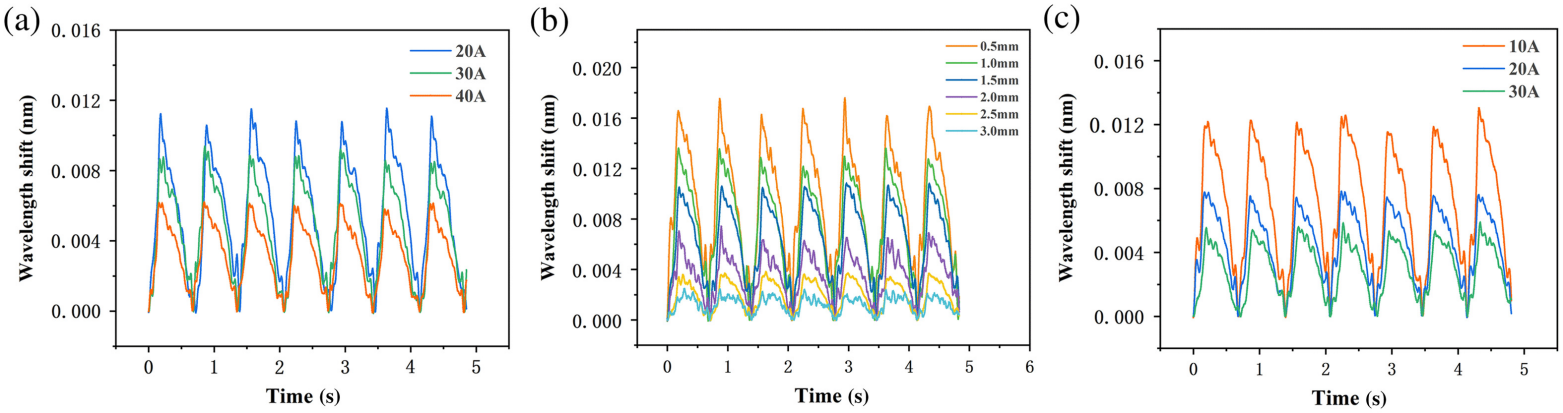
Measured Bragg wavelength shift of the FBG force sensor when conducting various gripping operations using the laparoscope, where panel (a) is the result for the phantom I with vessel of varying hardness, panel (b) is for the phantom II with varying thickness of tissue surrounding the vessels, and panel (c) is for the phantom III with varying hardness of silicone tissue surrounding the vessels.

Figure 5 displays the box plots of the results achieved for vessel phantoms I to III, illustrating a robust linear relationship between the mean PCFF values and the actual values of the variables across different phantoms. This correlation demonstrates the sensor’s high sensitivity and resolution in recognizing silicone blood vessels with different hardness, as well as those surrounded by tissues of varying thickness and hardness. Figure 6 presents a representative pulse waveform randomly selected from the gripping process of vessel phantoms I to III over a duration of 5 s. In vessel phantom I, the PCFF decreases with increasing hardness of the silicone blood vessel. In the vessel phantom II, having the same silicone blood vessel, the PCFF decreases as the thickness of the surrounding tissue increases. Similarly, in vessel phantom III, for a given silicone blood vessel, the PCFF decreases with the increasing hardness of the surrounding tissue.

### 3.2 Performance in Detecting Vessels in Porcine Models

As shown in Tables 4–6, in porcine vessel model I, where porcine abdominal arteries and veins were gripped, the mean PCFF values were 0.047 N and 0.022 N, respectively. The differences between these groups were statistically significant (p<0.001), indicating that the FBG-based sensor can distinguish between different porcine vessels. In porcine vessel model II, where arterial vessels surrounded by 1.0, 2.0, and 3.0 mm-thick adipose tissues were gripped, the mean PCFF values were 0.122 N, 0.066 N, and 0.046 N, respectively. These differences were also statistically significant (p<0.001), demonstrating that the FBG-based sensor can identify porcine vessels surrounded by varying tissue thicknesses. In porcine vessel model III, the mean PCFF values were 0.059 N and 0.032 N when gripping arterial vessels surrounded by adipose and muscle tissues, respectively. A t-test for independent samples showed a statistically significant difference between the two groups (p<0.001).

**Table 4:** Comparison of PCFF values of porcine abdominal arteries and veins.

	Mean of PCFF (N)	VS Abdominal vein (Z value)
Abdominal arteries	0.047	−10.945^***^
Abdominal vein	0.022	

***p<0.001.

**Table 5:** Comparison of inter-group averages of PCFF surrounding different thicknesses of tissue.

	Mean of PCFF (N)	VS 2.0 mm (H value)	VS 3.0 mm (H value)
1.0 mm	0.122	84.33^***^	154.70^***^
2.0 mm	0.066	—	70.38^***^
3.0 mm	0.046	—	—

***p<0.001.

**Table 6:** Comparison of inter-group averages of PCFF surrounding different hardness of tissue.

	Mean of PCFF (N)	VS Porcine muscle (T value)
Porcine adipose	0.059	−24.525^**^
Porcine muscle	0.032	—

**p<0.01.

**Fig. 7:**
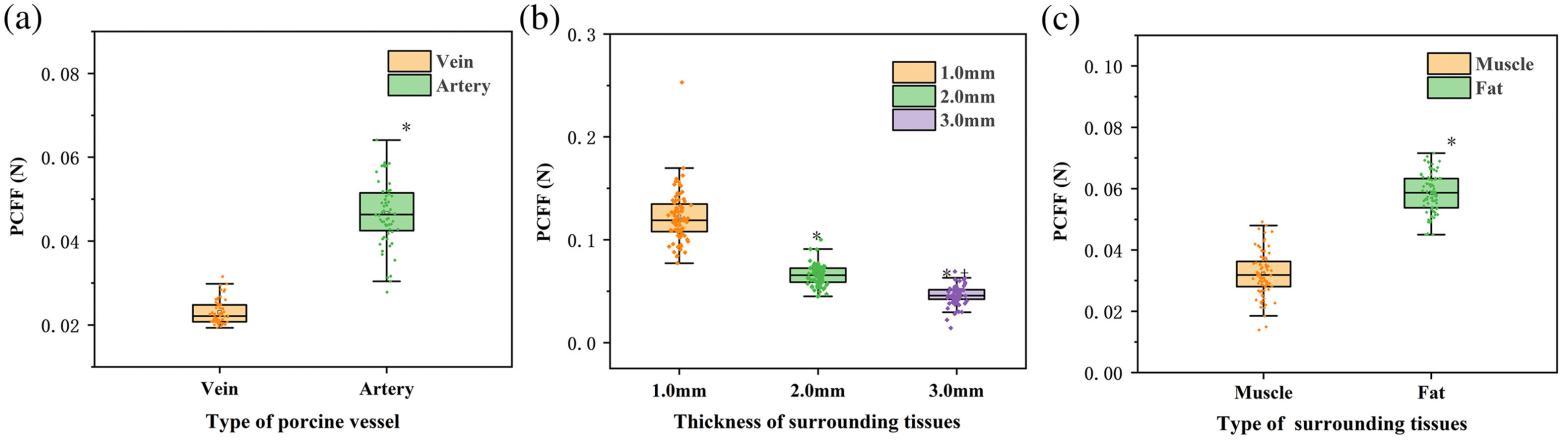
Box plots for PCFF results achieved when using laparoscope to grip the porcine vessel (a) model I with abdominal artery and vein, (b) model II with porcine carotid artery surrounded by adipose tissue having thicknesses of 1.0, 2.0, and 3.0 mm, and (c) model III with the porcine carotid artery surrounded by porcine musculature and adipose tissues.

**Fig. 8:**
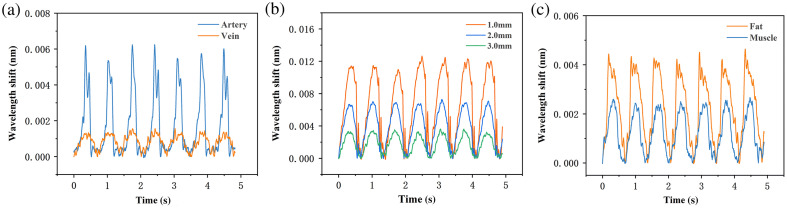
(a) Signals for gripping abdominal arteries and veins. (b) Signals for gripping tubes with varying thicknesses of porcine adipose tissues surrounding the porcine carotid arteries. (c) Signals for gripping tubes with porcine musculature and adipose tissues surrounding the porcine carotid arteries.

Figure 7 displays the box plots of the achieved results for porcine vessel models I to III. These results collectively demonstrate statistically significant differences among the groups, revealing that the laparoscopic grasper integrated with FBG tactile force sensors is capable of identifying vessels in various models based on the measured gripping forces. Figure 8 displays a typical pulse waveform randomly selected from the gripping process of porcine vessel models I, II, and III over 5-s duration. Specifically, in model I, the PCFF decreases when gripping the abdominal vein compared with the abdominal arteries. In model II, the PCFF decreases as the adipose tissue thickness increases. In model III, the PCFF decreases when the surrounding adipose tissue is replaced with porcine muscle.

### 3.3 Result of the Neural Network Model

**Fig. 9:**
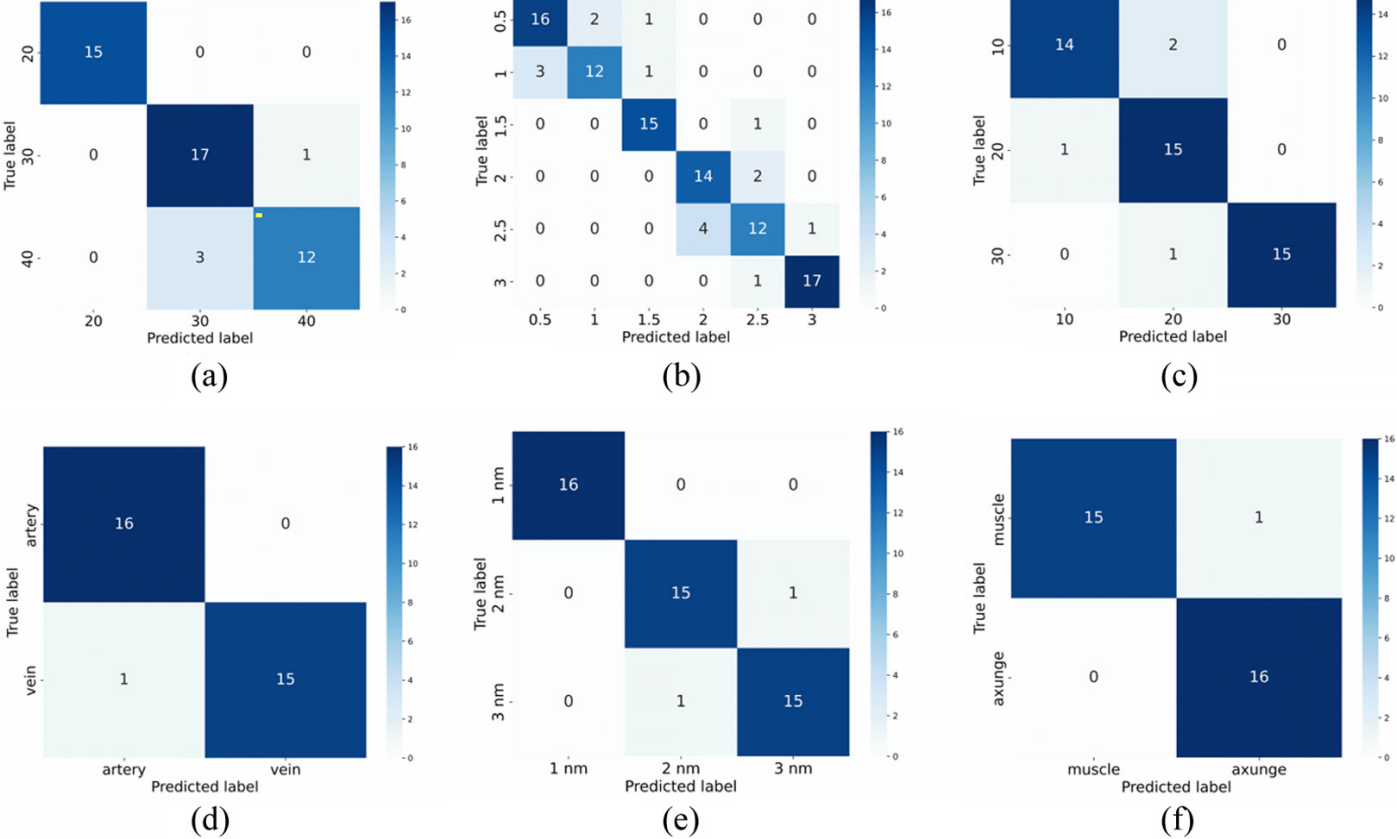
Confusion matrix of different tasks. (a) Vessel hardness classification, (b) thickness of perivessel tissue classification, (c) hardness of perivessel tissue classification, (d) porcine vessel classification, (e) thickness of perivessel tissue classification (porcine), (f) hardness of perivessel tissue classification (porcine).

To realize the identification and classification of various tissue vessels using the CNN-LSTM algorithm, a number of gripping forces were obtained by the FBG tactile force sensor on a laparoscopic grasper. The signal sequences collected from silicone phantoms I, II, and III were 245, 507, and 240, respectively, whereas for the porcine vessel phantoms I to III, the signal sequences of 160, 240, and 160 were recorded. To assess the performance of the CNN-LSTM algorithm, precision is calculated for the test dataset. As shown in Table 7, for classifying silicone vessels with varying hardness levels, the algorithm achieved a precision of 92.44%. When identifying silicone vessels surrounded by tissues of different thicknesses, the achieved precision was 84.23%. In silicone phantom III, the performance of the algorithm for classifying silicone vessels surrounded by tissues of different hardness levels was 92.22% in precision. For porcine abdominal arteries and veins classification, the algorithm achieved a precision of 97.06%. When identifying porcine vessels surrounded by tissues of varying thicknesses, the precision was 95.83%. In the porcine vessel model III, the model’s performance for classifying porcine vessels surrounded by different types of tissue was 97.06% in precision. As shown in Fig. 9, the confusion matrix provides a visual representation of the accuracy of the classification model, showing where the model is performing well.

**Table 7:** Precision of different classifying tasks.

Classifying performance of various tasks	Precision (%)
Hardness for silicone vessel	92.44
Thickness of tissue surrounding the silicone vessel	84.23
Hardness of tissue surrounding the silicone vessel	92.22
Porcine abdominal arteries and abdominal vein	97.06
Thickness of tissue surrounding the porcine vessel	95.83
Hardness of tissue surrounding the porcine vessel	97.06

## 4 Discussion

The objective of this study is to address the lack of force feedback in MIS, which poses challenges for surgeons in identifying blood vessels and increases the likelihood of unintended vessel damage. Based on the rhythmic and pulsatile nature of blood vessels, this work demonstrates a laparoscopic grasper integrated with an FBG-based tactile force sensor for blood vessel identification.

Initially, we constructed six types of *ex vivo* vessel simulation systems based on the human actual circulatory system and clinical scenarios. These models ranged from silicone representations to anatomically precise porcine vessel models, encompassing various vessel categories as well as the same vessel covered by different tissues. This comprehensive approach allowed for a transition from qualitative to quantitative analysis. By repeatedly grasping these vessel models using the laparoscopic grasper integrated with FBG sensors, we recorded the force signals and established a comprehensive database. The results demonstrated statistically significant differences in mean PCFF among groups across various clinical needs, highlighting the potential of the laparoscope with a sensor in diverse scenarios.

To expedite blood vessel identification, the CNN-LSTM algorithm is proposed for signal processing. As a result, the neural network algorithm for the six vascular models exhibited a great precision rate. This confirms that the intelligent laparoscopic grasper, equipped with the FBG sensor and combined with the CNN-LSTM algorithm, can achieve accurate and real-time blood vessel identification in *ex vivo* vascular models.

Compared with existing approaches utilizing fluorescence imaging, ultrasound, and piezoelectric sensors for blood vessel identification, FBG-based tactile force sensors offer advantages such as high biocompatibility and small size, effectively overcoming the low biocompatibility and complex system issues associated with current research methods. Furthermore, the vessel models constructed in this work, ranging from *ex vivo* silicone vessels to porcine vessels and incorporating various tissues surrounding the vessels, enhance clinical relevance. The mean PCFF value showed a strong correlation with blood vessels in different scenarios, and the statistical significance of inter-group differences across various clinical situations indicates the sensor’s ability to accurately identify blood vessels in diverse applications.

The goal of the neural network algorithm demonstrated in this work is to analyze the Bragg wavelength of FBG sensors to classify and predict the positions of vessels under different conditions. The CNN-LSTM hybrid architecture was chosen to address the dual spatiotemporal characteristics of FBG tactile force signals in laparoscopic surgery. The CNN branch, utilizing 1D convolutions and bottleneck blocks, extracts morphological patterns from localized force variations, such as short-duration pulses during vessel contact. Its residual structure helps mitigate gradient vanishing and preserves transient features. By contrast, the LSTM branch captures long-range temporal dependencies, such as periodic pulsation rhythms or phases of grasper movement, which are not precisely retrieved by using a pure CNN. The combination of these two pathways facilitates joint spatiotemporal reasoning, which is essential for distinguishing vessels with similar magnitudes but different dynamic behaviors, such as arteries and veins. By combining CNNs and LSTMs, we leverage CNN’s strength in local feature extraction and LSTM’s capability to process temporal information, resulting in a more comprehensive signal analysis and reduced risk of overfitting. From the test results for the six vascular models, good precision is achieved, indicating that integrating the CNN-LSTM algorithm significantly enhances the accuracy of blood vessel identification.

Although the current research setup is grounded in real clinical scenarios, certain limitations persist in practical clinical applications. The current *in vitro* experiments utilize water as the circulating fluid, which however differs significantly from human blood in terms of density, viscosity, and the complex microenvironment that includes blood cells. This limitation prevents an accurate simulation of blood’s physiological properties. To address this, further experiments will incorporate mixtures of water and glycerol at various ratios, or animal blood, to better simulate conditions such as diabetes, hypertension, and hyperlipidemia. These adjustments will allow for more realistic *in vitro* circulation studies and a deeper exploration of characteristic pulsation patterns. Moreover, clamping techniques in clinical practice are highly varied, which are undertaken to enrich data analysis across a range of clamping modes, including different angles, vascular morphologies, and more diverse clinical scenario simulations, thereby expanding the database. As the dataset grows, the model will be retrained using an expanded range of clinical scenarios, larger data volumes, and data from *in vivo* animal or human experiments. This will enhance the model’s training outcomes and improve its ability to generalize across a broader array of clinical situations. The ultimate goal is to develop an intelligent laparoscopic vessel identification system that integrates FBG force sensors to provide the surgeons with predicted vascular information as real-time feedback, offering critical intraoperative data on vessel location and aiding in vessel localization. This work aims to provide valuable insights and guidance for the future development of intelligent medical devices and surgical assistance systems.

## 5 Conclusion

In a word, in this work, an intelligent laparoscopic grasper integrated with an FBG tactile force sensor is demonstrated to perform gripping operations on various vascular models. The approach employs a combination of a CNN-LSTM algorithm for signal recognition. Statistically significant variations in gripping force are observed across various scenarios, and the CNN-LSTM algorithm exhibits high precision in identifying blood vessels in different settings. The promising results indicate that the laparoscopic grasper integrated with FBG sensors can accurately identify blood vessels, showing high potential for vessel identification during MIS.

## Data Availability

The datasets used and analyzed during the current study are available from the corresponding author upon reasonable request.

## References

[1] Pérez Ayme A. P. et al., “Advancements in minimally invasive surgical techniques: a comprehensive review,” Salud, Cienc. Tecnol. 4, 745 (2024). 10.56294/saludcyt2024745

[2] Pasupuleti S. et al., “Role of robotic systems in minimally invasive surgery: benefits, risks, and future directions,” Int. J. Sci. Res. Eng. Manage. 5, 1–6 (2021). 10.55041/IJSREM6812

[3] Enayati N. et al., “Haptics in robot-assisted surgery: challenges and benefits,” IEEE Rev. Biomed. Eng. 9, 49–65 (2016). 10.1109/RBME.2016.2538080 26960228

[4] Wottawa C. R. et al., “Evaluating tactile feedback in robotic surgery for potential clinical application using an animal model,” Surg. Endosc. 30, 3198–3209 (2016). 10.1007/s00464-015-4602-2 26514132 PMC4851934

[5] Hsu C. S. et al., “Laparoscopic modified simple ureteroneocystomy in iatrogenic lower third ureter injury during gynecology surgery,” Taiwan J. Obstet. Gynecol. 63, 777–780 (2024). 10.1016/j.tjog.2024.04.019 39266165

[6] Poston C. M. et al., “Gastrointestinal and genitourinary complications of gynecologic laparoscopic surgery,” Curr. Obstet. Gynecol. Rep. 14, 10 (2025). 10.1007/s13669-025-00414-4

[7] Khan A. F. et al., “Tissue stress from laparoscopic grasper use and bowel injury in humans: establishing intraoperative force boundaries,” BMJ Surg. Interv. Health Technol. 3, e84 (2021). 10.1101/2021.02.19.21252109 PMC8749288 35047803

[8] Tinelli R. et al., “Left external iliac vein injury during laparoscopic pelvic lymphadenectomy for early-stage ovarian cancer: our experience and review of literature,” Front. Surg. 9, 843641 (2022). 10.3389/fsurg.2022.843641 35356499 PMC8959709

[9] Buras A. L. et al., “Major vascular injury during gynecologic cancer surgery,” Gynecol. Oncol. Rep. 37, 100815 (2021). 10.1016/j.gore.2021.100815 34258355 PMC8259293

[10] Sallam A. et al., “Impact of concomitant vascular injury on the outcome of bile duct injury,” Egypt. J. Surg. 42, 502–508 (2023). 10.4103/ejs.ejs_64_23

[11] Takahashi J. et al., “The introduction of fluoroscopic surgery: a report of an initial trial case,” Int. J. Surg. Case Rep. 115, 109202 (2024). 10.1016/j.ijscr.2023.109202 38277985 PMC10837057

[12] Yamauchi S. et al., “Indocyanine green and near-infrared fluorescence imaging in minimally invasive gastric cancer surgery: a narrative review,” Mini-Invas. Surg. 27, 185–197 (2023). 10.7602/jmis.2024.27.4.185 PMC11651914 39675751

[13] Ciocan R. A. et al., “Robot-guided ultrasonography in surgical interventions,” Diagnostics 13, 2456 (2023). 10.3390/diagnostics13142456 37510199 PMC10378616

[14] Türk Y. et al., “The role of multislice computerized tomography angiography in assessing postoperative vascular complications in liver transplant patients,” Turk. J. Med. Sci. 49, 1212–1220 (2019). 10.3906/sag-1902-145 31408295 PMC7018385

[15] Li C. et al., “Smart blood vessel detection system for laparoscopic surgery,” IEEE J. Transl. Eng. Health Med. 10, 2500207 (2022). 10.1109/JTEHM.2022.3159095 35345534 PMC8939714

[16] Beasley R. A. et al., Tactile Tracking of Arteries in Robotic Surgery, pp 3801–3806, IEEE (2002).

[17] Jung S. et al., “Noninvasive flow monitoring in simple flow phantom using resistive strain sensors,” Sensors 21, 2201 (2021). SNSRES 0746-9462 10.3390/s21062201 33801114 PMC8004077

[18] Zhu X. et al., “Advancements in fiber optic tactile sensors: a comprehensive review on principles, fabrication, and applications,” Opt. Laser Eng. 186, 108777 (2025). 10.1016/j.optlaseng.2024.108777

[19] Gunawardena D. S. et al., “Polymeric fiber sensors for insertion forces and trajectory determination of cochlear implants in hearing preservation,” Biosens. Bioelectron. 222, 114866 (2023). BBIOE4 0956-5663 10.1016/j.bios.2022.114866 36463651

[20] Ahmadi R. et al., “Micro-optical force distribution sensing suitable for lump/artery detection,” Biomed. Microdevices 17, 10 (2015). 10.1007/s10544-015-9931-3 25653070

[21] Ho S. C. M. et al., “Fiber Bragg grating based arterial localization device,” Smart Mater. Struct. 26, 65020 (2017). SMSTER 0964-1726 10.1088/1361-665X/aa6ec2

[22] Li T. et al., “Reaction force mapping by 3-axis tactile sensing with arbitrary angles for tissue hard-inclusion localization,” IEEE Trans. Biomed. Eng. 68, 26–35 (2021). IEBEAX 0018-9294 10.1109/TBME.2020.2991209 32396067

[23] Wang P. et al., “Smart laparoscopic grasper integrated with fiber Bragg grating based tactile sensor for real‐time force feedback,” J. Biophotonics 15, e202100331 (2022). 10.1002/jbio.202100331 35020276

[24] Huang X. et al., “An intelligent grasper to provide real-time force feedback to shorten the learning curve in laparoscopic training,” BMC Med. Educ. 24, 161 (2024). 10.1186/s12909-024-05155-1 38378608 PMC10880316

[25] Magkoutas K. et al., “Continuous monitoring of blood pressure and vascular hemodynamic properties with miniature extravascular hall-based magnetic sensor,” JACC Basic Transl. Sci. 8, 546–564 (2023). 10.1016/j.jacbts.2022.12.008 37325404 PMC10264706

[26] Ma Z. et al., “Smart vascular grafts with integrated flow biosensors for hemodynamic real-time monitoring and vascular healthcare,” ACS Nano 19, 7661–7676 (2025). ANCAC3 1936-0851 10.1021/acsnano.4c09980 39818734

[27] Zhong Q. et al., “Cluster non-Gaussian functional data,” Biometrics 77, 852–865 (2021). BIOMB6 0006-341X 10.1111/biom.13349 32749677

[28] Ba D. P. K. J., “Adam: a method for stochastic optimization,” in Conf. Pap. at ICLR 2015 (2015).

[29] Mahardika T. N. et al., “PPG signals-based blood-pressure estimation using grid search in hyperparameter optimization of CNN-LSTM,” Diagnostics 13, 2566 (2023). 10.3390/diagnostics13152566 37568929 PMC10417316

[30] Selvasingham S. et al., “Classifying numbers from EEG data—which neural network architecture performs best?” Stud. Health Technol. Inf. 292, 103–106 (2022). SHTIEW 0926-9630 10.3233/SHTI220333 35575857

